# Neurocognitive improvement in HIV-positive patients treated with dolutegravir-based regimens

**DOI:** 10.4102/sajpsychiatry.v29i0.2071

**Published:** 2023-09-28

**Authors:** Janine Rodrigues, Karishma Lowton

**Affiliations:** 1 Department of Psychiatry, Faculty of Medicine, University of the Witwatersrand, Johannesburg, South Africa

## Abstract

**Background:**

Neurocognitive disorders due to human immunodeficiency virus (HIV) remain highly prevalent, specifically mild forms despite effective antiretroviral therapy (ART). Dolutegravir-based regimens are the first line of treatment for adult HIV-positive patients. Controversies exist regarding the neurocognitive effects of dolutegravir. Evidence regarding the neurocognitive effects of dolutegravir is important, in support of its use in patients with HIV-associated neurocognitive disorders (HAND).

**Aim:**

This study aimed to describe the change in cognitive function using the International HIV Dementia Scale (IHDS) and Brief Neuropsychological Cognitive Examination (BNCE) in HIV positive, treatment naïve patients before and 3 months after initiation of ART using a dolutegravir-based regimen.

**Setting:**

The HIV initiation clinic of Hillbrow Community Health Centre in Johannesburg.

**Methods:**

This prospective, quantitative cohort study assessed adult HIV-positive patients who were ART naïve being initiated on a dolutegravir-based regimen, using the BNCE and IHDS at baseline and after 3 months of treatment.

**Results:**

Neurocognitive test results of 26 participants showed significant improvements for IHDS (*Z* = 1.84, *p* = 0.033) and time to complete BNCE (*Z* = 2.47, *p* = 0.007). BNCE total results showed improvements that were not significant (*Z* = 1.44, *p* = 0.075); however, Part 2 of the BNCE reflecting that of executive function showed significant improvements (*Z* = 66.5, *p* = 0.043).

**Conclusion:**

The trend of neurocognitive function is towards improvement in HIV-positive treatment naïve patients who receive 3 months of dolutegravir-based ART.

**Contribution:**

The findings support the use of dolutegravir-based regimens in the treatment of patients with HIV-associated neurocognitive disorders.

**Keywords:**

HIV-associated neurocognitive disorders; BNCE; IHDS; dolutegravir; neurocognitive screening; neurocognitive impairment; South Africa.

## Introduction

Human immunodeficiency virus (HIV) infection leading to acquired immunodeficiency syndrome (AIDS) remains a common cause of neurocognitive impairment despite the increasing availability and use of effective antiretroviral therapy (ART).^1^ While HIV-associated neurocognitive disorders (HAND) are highly prevalent in both developed and developing countries, approximately 72% of the world’s cases are found in sub-Saharan Africa.^2,3^

Viral invasion of the central nervous system (CNS) occurs early in the course of infection, affecting subcortical areas of the brain.^2,4,5^ The dysregulated CNS immune response and consequent low-grade neuroinflammation persists despite the use of ART and is hypothesised as the main contributing factor towards the development of HAND.^6^ Other contributing factors include advancing age, male gender, lower cluster of differentiation 4 (CD4) count, lower levels of education and drug or alcohol abuse.^7^ These factors, as well as comorbid conditions such as depression, may both co-exist and act synergistically, contributing towards the severity of neurocognitive dysfunction.^7,8^ Furthermore, the HIV-1 subtype Clade C has been identified as the predominant subtype in South Africa and a risk for the development of HAND.^4^

The Frascati criteria are commonly used to categorise HAND based on severity.^9^ The spectrum ranges from milder forms including asymptomatic neurocognitive impairment (ANI) and mild neurocognitive disorder (MND), to severe in HIV-associated dementia (HAD).^9^ Areas of cognitive impairment involve attention, working memory and executive function with amnestic disturbances not commonly occurring early in the course of the illness.^10^ Other characteristic features include motor slowing and impaired processing speed.^5^ Cognitive impairment can lead to social and occupational dysfunction, poor treatment adherence, unsafe sexual practices, decreased employment and substance abuse, ultimately resulting in impaired quality of life.^2,5^

While the introduction of ART has approximately halved the incidence of HAD, cognitive dysfunction in milder forms is persistent and increasing in prevalence in patients receiving virologically suppressive ART regimens.^4,5,11^ A recent meta-analysis found that an estimated 43.9% of people living with HIV (PLWH) were affected by HAND, with 79% of these consisting of ANI or MND.^12^ This may be related to the direct and indirect effects of the virus on the CNS and potential neurotoxic effects of ART.^4^ The penetrability of the blood brain barrier (BBB) also impacts on ART efficacy in the prevention and treatment of HAND.^4^ Penetration effectiveness may impact neurocognitive function with viral suppression in cerebrospinal fluid (CSF) correlating with improved cognition.^11^ Further cognitive benefits arise from immune recovery and decreased immune activation.^11^ CNS penetration effectiveness (CPE) ranking systems grade ART regimens based on their ability to cross the BBB and achieve control of CNS viral replication.^13^ While higher CPE regimens are associated with improved CSF viral suppression, they could also lead to neurotoxicity due to high drug penetrance in the CNS.^4^ Despite mixed results in studies correlating higher CPE regimens with neurocognitive function, evidence supports that early ART initiation improves cognition and also identifies a ‘therapeutic window’ in which HAND may be prevented.^4,13^ Factors preserving cognitive function in HIV-infected individuals include early diagnosis, ART initiation within 6 months of seroconversion and reduction of the inflammatory cascade following HIV infection.^13^

According to the National Department of Health 2019 ART clinical guidelines, the first-line ART regimen recommended for adults is a combination of tenofovir, lamivudine and dolutegravir.^14^ The World Health Organization (WHO) also recommends dolutegravir-based regimens as first line.^15^ Dolutegravir was compared to efavirenz in the SINGLE trial and its benefits included superiority, better tolerability and decreased resistance profile.^16^ Despite its growing use worldwide, there is some concern around CNS safety due to reported neuropsychiatric adverse events (NP-AEs).^13^ In some studies, the rate of dolutegravir discontinuation because of NP-AEs was 1% – 6%, with common complaints including insomnia and depression.^15^ With regard to neurocognitive function, the difference pre- and post-dolutegravir initiation is yet to be defined.^15^ Despite concerns and conflictual evidence, studies support the CNS safety of initiating and switching to a dolutegravir-containing ART regimen.^13^

In diagnosing HAND, patients should undergo neuropsychological, psychiatric and medical evaluation.^7^ The neuropsychological assessment should include a comprehensive, standardised neuropsychological protocol that covers multiple domains of cognitive function.^9^ This can be time consuming and challenging especially in resource-limited settings.^9^ Screening tests such as the Folsteins mini mental state exam (MMSE) and the International HIV Dementia Scale (IHDS) are commonly used to detect HAND; however, when used in isolation may lack sensitivity and reliability in detecting mild neurocognitive impairments.^9^ Factors that could affect outcome of assessments and require consideration include age, language, culture, level of education and comorbidities.^5^ Construct validity for use of neuropsychological tests in South Africa, specifically in isiXhosa speakers, is yet to be confirmed.^5^ However, evidence confirms that the IHDS has sensitivity of 45% and specificity of 79% in identifying severe HAND in the South African context.^5^ The Brief Neuropsychological Cognitive Examination (BNCE) is another screening test, which aims to rapidly assess a range of cognitive functions.^17^ It assists in assessing the patients ability to live independently as well as monitoring disease progression or improvement with treatment.^18^

Due to the ongoing high incidence of mild neurocognitive dysfunction despite ART and the controversies that exist regarding dolutegravir’s effect on neurocognitive function, more research is needed to further understand this relation, given its recent addition to South Africa’s first-line regimen.

## Aim and objectives

The primary aim of this study was to describe the change, if any, in cognitive function using the IHDS and BNCE in HIV-positive treatment-naïve patients, before and after initiation of treatment with 3 months of ART using a dolutegravir-based regimen. It was hypothesised that results would show improvement. Secondary objectives included to compare patient sociodemographic data (age, gender, race and level of education) as well as blood results, specifically baseline CD4 count and viral load (VL) measured at 3 months post-ART initiation and correlate these findings to change in neurocognitive results.

## Research methods and design

### Study design and setting

This study was a prospective, quantitative and descriptive cohort study. It was conducted between June 2021 and July 2022 at the HIV initiation clinic at Hillbrow Community Health Centre in Johannesburg. This unit falls under the district level of care in the public health sector of the Gauteng Department of Health in South Africa.

### Study population and sampling

The study sample population comprised English-speaking and consenting, adult HIV-positive patients who were ART naive being initiated on a dolutegravir-containing regimen. Exclusion criteria consisted of any previously diagnosed neurocognitive disorder or the presence of other known causes of neurocognitive impairment such as depression, stroke, intellectual disability, traumatic brain injury and current or recent substance use. Patients older than 60 years were excluded from the study to preclude age-appropriate neurocognitive decline as a confounding factor. The calculated required sample size was 33 participants to achieve significant results with a certainty of 95%. The intended sample size was 50 participants to account for participant dropout and other unforeseen circumstances. Patients attending the HIV initiation clinic were chosen by stratified sampling. Adult patients who were ART naïve and to start a dolutegravir-containing regimen were given information regarding the study by use of a pamphlet and provided written consent to participate in various aspects of the study. A screening questionnaire was then provided comprising inclusion and exclusion criteria. Eligible participants were allocated a participant number and included in the study.

### Intervention and data collection

The primary investigator collected and documented demographic information and assessed participants using the IHDS and BNCE. The IHDS screening tool is a three-part examiner administered test comprising of a non-dominant finger-tapping test, a non-dominant Luria hand sequence and a four-word recall test.^5^ The maximum score is 12 with scores of 11 or 12 considered normal and scores of 10 or lower indicating need for further neuropsychological testing. The second test, the BNCE, is made up of two parts with five subtests in each part. Part 1 subtests are mainly related to conventional types of information processing (e.g. naming body parts, orientation) and Part 2 subtests are aimed at assessing the performance of executive functioning.^17,18^ Specific to the South African population, modification of Part 1, section B of the BNCE titled presidential memory was required, in order to be appropriate to the knowledge and culture of the study group. Questions were adapted from assessing presidential memory of the United States to that of African presidential history that is well known to the people of South Africa. This maintains a culturally fair and appropriate measure of long-term memory and crystalised intelligence, in the absence of other culturally appropriate tests. The maximum score is 30 with total scores of 27 or less indicating cognitive impairment. Scores are then further divided into mild (22–27), moderate (10–21) and severe (0–9).^17,18^ The time taken to complete the BNCE was also considered in evaluating cognitive improvement. This is not a requirement for the administration of the BNCE, however, is useful when assessing processing speed in the consultation.^18^ Neurocognitive testing was conducted on the day of ART initiation and repeated after approximately 3 months after confirming treatment adherence. Blood results were documented, specifically CD4 count at baseline and VL after 3 months of receiving ART.

### Data analysis

Of the 63 participants that completed the screening questionnaire, data from a total of 26 participants were used in the analyses. These participants completed the 3-month follow-up to produce useable data to address the aims of the study. The data were collated in Microsoft Excel™ and statistically analysed in R software (version 4.00; www.R- project.org).

The data set was screened for departure from normality using Shapiro-Wilk tests. Non-parametric analyses were conducted, including Spearman regressions, Pearson chi-squared analyses (with binary post hocs), logistic regressions for dichotomous outcomes, general linear regressions and Wilcoxon matched pairs tests (with continuity correction for low values, as necessary). Some analyses were one-tailed based on directional hypotheses (e.g. Wilcoxon tests), and all other analyses were two-tailed. Model alpha was set at 0.05. Data are reported as counts and percentages (categorical data) or mean and standard deviation (continuous data) and presented descriptively in the text, tables and figures.

### Ethical considerations

Ethics clearance was obtained from the University of Witwatersrand Human Research Ethics Committee (Clearance Certificate No. M201102). All procedures performed were in accordance with the 1964 Helsinki Declaration and it’s later amendments.^19^ Approval was also granted by The National Health Research Database (Ref: GP_202008_151). Written consent was obtained from all individual participants involved in the study. Confidentiality was maintained by the removal of identifiable information and use of participant numbers.

## Results

**FIGURE 1:**
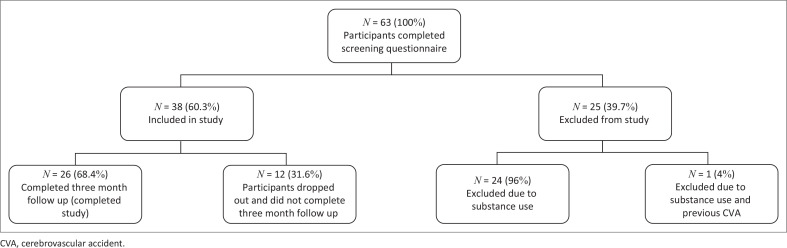
Participants in study.

Of the 63 patients screened for participation, 38 were eligible. Excluded participants comprised mostly males and the majority due to substance use. After a loss to follow up drop out rate of 31, 6%, 26 participants completed the study (Figure 1). The days from baseline to 3 month follow-up ranged from 70 to 103 with a mean of 83.7 days.

The demographic data as depicted in Table 1 showed that the study population had a mean age of 36.5 (s.d. = 7.1) years, there was significantly more females (χ^2^ = 9.85, *df* = 1, *p* = 0.002) and all were black African ethnicity. The years of formal education ranged from 7 to 14 years with a mean of 11.0 (s.d. = 1.7) years. CD4 counts were found to have a mean of 304.1 (s.d. = 228.2) cells/mm^3^ and when categorised into below or above counts of 200 cells/mm^3^, the difference was not significant (χ^2^ = 1.00, *df* = 1, *p* = 0.317). The viral load measured at 3 months varied with significantly more patients showing virological suppression at levels of 0–20 (χ^2^ = 8.83, *df* = 3, *p* = 0.032) (Table 1).

**TABLE 1:** Demographics and clinical data of study participants.

Variables	Values				Statistics*		
	Mean	s.d.	*n*	%	*χ* ^2^	*df*	*p*-value
Age (years) (*N* = 26)	36.5	7.1	-	-	-	-	-
**Gender (*N* = 26)**	-	-	-	-	**9.85**	**1**	**0.002**
Female	-	-	21	80.8	-	-	-
Male	-	-	5	19.2	-	-	-
**Race (*N* = 26)**	-	-	-	-	-	-	-
Black people	-	-	26	100	-	-	-
Education (years) (*N* = 26)	11.0	1.7	-	-	-	-	-
**Baseline CD4 (*N* = 25)**	-	-			1.00	1	0.317
≤ 200	-	-	10	40.0	-	-	-
> 200	-	-	15	60.0	-	-	-
**VL at 3 months (*N* = 23)**	-	-	-	-	**8.83**	**3**	**0.032**
< 20	-	-	11	47.8	-	-	-
21–100	-	-	5	21.7	-	-	-
101–500	-	-	6	26.1	-	-	-
> 500	-	-	1	4.3	-	-	-

Note: significant outcomes are shown in bold.

CD4, cluster of differentiation 4; s.d., standard deviation; VL, viral load.

* Pearson chi-squared for categorical data.

**FIGURE 2:**
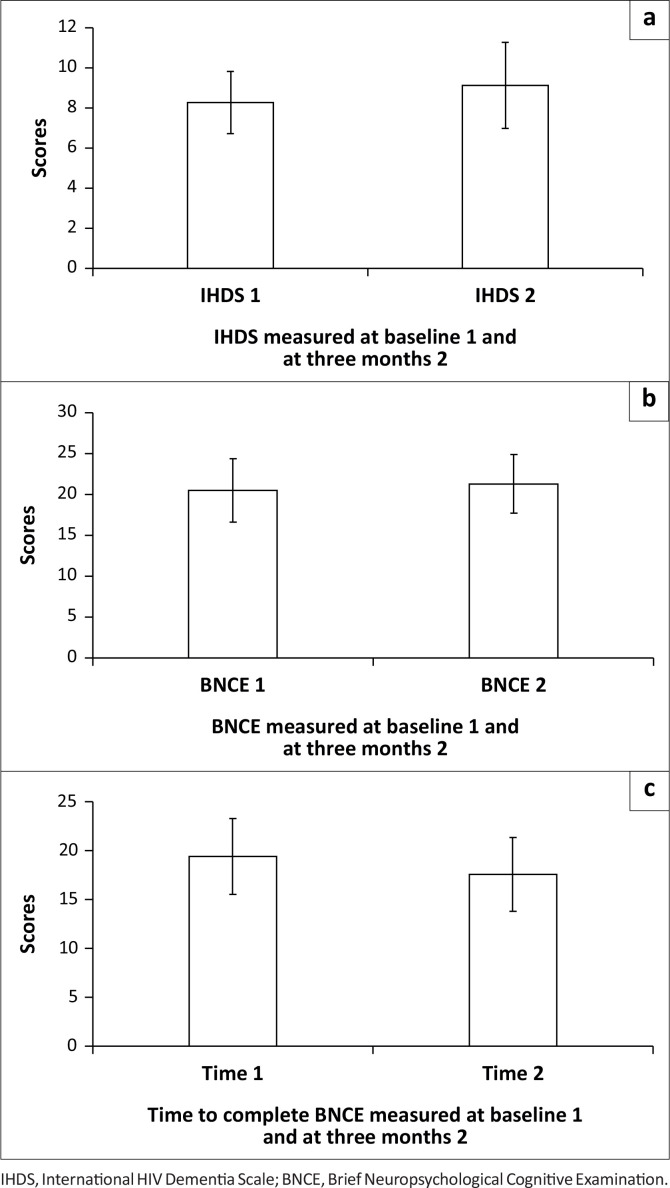
Mean (standard deviation) values of baseline and three months later (a) International HIV Dementia Scale, (b) Brief Neuropsychological Cognitive Examination total and (c) Brief Neuropsychological Cognitive Examination time intervals values.

The neurocognitive tests at baseline and at 3 months showed that IHDS results increased significantly from a baseline mean of 8.3 (s.d. = 1.6) to a 3 month mean of 9.1 (s.d. = 2.1) (Wilcoxon test: *Z* = 1.84, *p* = 0.033) (Figure 2). The BNCE total scores also increased from a baseline mean of 20.5 (s.d. = ± 3.9) to a 3 month mean of 21.3 (s.d. = ± 3.6), but the change was not statistically significant (Wilcoxon test: *Z* = 1.44, *p* = 0.075) (Figure 2). In contrast, BNCE time decreased significantly from baseline of 20.2 minutes (s.d. = ± 4.0) to a 3 month mean of 18.3 min (s.d. = ± 3.9) (Wilcoxon test: *Z* = -2.47, *p* = 0.007) (Figure 2).

When comparing the two parts of the BNCE, it was found that Part 2 scores were significantly lower than Part 1 scores for both BNCE 1 (Wilcoxon matched pairs test: *Z* = 297.5, *p* < 0.001) and BNCE 2 (Wilcoxon matched pairs test: *Z* = 262.0-, *p* < 0.001) (Table 2). At baseline, more patients scored six points lower in Part 2 than in Part 1 when compared with those scoring three to five points lower in Part 2 than in Part 1. The opposite is seen in BNCE scores at 3 months (Table 2). When comparing the change in Part 1 and Part 2 scores from baseline to 3 months, Part 2 scores showed higher scores that were clinically significant (*Z* = 66.5, *p* = 0.043) (Table 2).

**TABLE 2:** Comparison between Brief Neuropsychological Cognitive Examination at baseline (Brief Neuropsychological Cognitive Examination 1) and 3 months later (Brief Neuropsychological Cognitive Examination 2), specifically comparing Part 1 and Part 2 of each.

Cognitive variables	Baseline (BNCE 1)				At 3 months (BNCE 2)				Statistics*	
	Mean	s.d.	*n*	%	Mean	s.d.	*n*	%	*Z*	*p*-value
BNCE Part 1	11.88	1.84	-	-	11.70	1.51	-	-	110.5	0.425
BNCE Part 2	8.65	2.71	-	-	9.54	2.58	-	-	**66.5**	**0.043**
BNCE Part 2 lower than Part 1	-	-	22	84.6	-	-	19	73.1	-	-
BNCE Part 2 6 points lower than Part 1	-	-	7	26.8	-	-	2	7.7	-	-
BNCE Part 2 3 – 5 points lower than Part 1	-	-	2	7.7	-	-	10	38.5	-	-

Note: significant outcomes are in bold.

BNCE, Brief Neuropsychological Cognitive Examination; s.d., standard deviation.

* Wilcoxon paired comparison test (one-tailed) for statistical significance of mean improvement.

The relationship between socio-demographic variables and cognitive findings revealed some statistically significant correlations (Table 3). With every year of increasing age, the BNCE total score at 3 months decreased by 0.504 points (*p* = 0.009). The BNCE score at baseline also decreased with every year of increasing age; however, this was not found to be significant (*p* = 0.065). Years of education were found to be a significant predictor of both IHDS and time taken to complete BNCE. A one year increase in years of education increased the IHDS 1 and 2 scores significantly (*p* = 0.030 and *p* = 0.019, respectively). Similarly, with every 1 year increase in years of education, the time taken to complete the BNCE decreased both at baseline and 3 months later (*p* = 0.001 and *p* = 0.002, respectively). While BNCE total scores at baseline and 3 months were also found to increase with every year of education, this was not found to be significant (*p* = 0.061 and *p* = 0.075, respectively). The correlation between socio-demographic variables and improvement in neurocognitive test scores was not found to be significant (Table 3).

**TABLE 3:** Correlations between age, gender and years of education and International HIV Dementia Scale, Brief Neuropsychological Cognitive Examination and Brief Neuropsychological Cognitive Examination time and their differences.

Cognitive variables	Age*		Gender*		Years of education*	
	R	P	Estimate	P	*r*	*P*
IHDS 1	-0.099	0.631	0.332	0.397	**0.436**	**0.030**
IHDS 2	-0.088	0.669	0.024	0.920	**0.453**	**0.019**
IHDS 2 – IHDS 1	-0.031	0.881	-0.127	0.599	0.093	0.649
BNCE 1	-0.368	0.065	0.107	0.418	0.373	0.061
BNCE 2	**-0.504**	**0.009**	0.108	0.445	0.355	0.075
BNCE 2 – BNCE 1	-0.154	0.452	-0.032	0.869	-0.060	0.772
Time 1	0.386	0.093	0.039	0.805	**-0.700**	**0.001**
Time 2	0.413	0.069	0.270	0.135	**-0.652**	**0.002**
Time 2 – Time 1	0.038	0.871	0.386	0.122	0.226	0.337

Note: Significant outcomes are in bold.

IHDS, International HIV Dementia Scale; BNCE, Brief Neuropsychological Cognitive Examination.

* Spearman regression (age, years of education) and logistic regression (gender) for cognitive variables.

Participants with a baseline CD4 count of < 200 cells/mm^3^ were found to show a significant increase in BNCE scores (*p* = 0.045) and decrease in time taken to complete the BNCE (*p* = 0.015). In comparison, those with CD4 counts of > 200 cells/mm^3^ showed no significant change in IHDS, BNCE and time scores (*p* = 0.104, *p* = 0.327 and *p* = 0.164, respectively) (Table 4).

**TABLE 4:** Baseline cluster of differentiation 4 and improvement in cognitive function.

Baseline CD4	Cognitive variables																	
	IHDS						BNCE						Time					
	1		2		*Z* *	*p*-value*	1		2		*Z* *	*p*-value*	1		2		*Z* *	*p*-value*
	Mean	s.d	Mean	s.d.			Mean	s.d	Mean	s.d.			Mean	s.d	Mean	s.d.		
CD4 < 200 (*N* = 10)	8.1	1.8	9.2	2.3	-1.49	0.084	20.4	4.6	22.2	3.7	**-1.74**	**0.045**	21.4	4.7	18.6	3.2	**2.21**	**0.015**
CD > 200 (*N* = 15)	8.4	1.5	9.1	2.2	-2.75	0.104	20.4	3.5	20.7	3.6	-2.85	0.327	18.4	4.5	19.0	3.4	3.75	0.164

Note: Significant outcomes are in bold.

IHDS, International HIV Dementia Scale; BNCE, Brief Neuropsychological Cognitive Examination; CD4, cluster of differentiation 4.

* Wilcoxon paired comparison test (one-tailed) for statistical significance of mean improvement

No significant correlation was found between VL measured at 3 months and improvement in neurocognitive test results (Table 5).

**TABLE 5:** The relationship between viral load and differences in International HIV Dementia Scale, Brief Neuropsychological Cognitive Examination and Brief Neuropsychological Cognitive Examination time.

Cognitive variables	Viral load (statistics*)		
	*χ* ^2^	*df*	*p*-value
IHDS 2 – IHDS 1	6.49	3	0.090
BNCE 2 – BNCE 1	6.77	3	0.080
Time 2 – Time 1	6.43	3	0.092

Note: None of the outcomes were statistically significant.

IHDS, International HIV Dementia Scale; BNCE, Brief Neuropsychological Cognitive Examination.

* General linear for cognitive variables.

## Discussion

This study aimed to describe the changes in cognitive function at baseline and 3 months after initiation of ART using a dolutegravir-based regimen with the hypothesis that results would show improvement. Significant improvements were noted in both IHDS scores and time taken to complete the BNCE. This supports improvement in subcortical neurocognitive function tested in the IHDS, as well as processing speed indicated by the time taken to complete the BNCE. While the total BNCE scores did show improvements from baseline, these results were not found to be significant. This is consistent with findings from a study done in 2020 assessing change in cognitive function over a 6 month period of using dolutegravir as assessed by the Montreal Cognitive Assessment (MoCA).^20^ The authors concluded that while 6 months was long enough to see VL suppression in both plasma and CSF, this may not be enough time to confirm change in cognitive function.^20^ The time factor may explain the findings in that not enough time was allowed for all aspects of cognition to improve between testing. Cysique et al. found that cognitive improvement can start as early as 12 weeks; however, it occurs more often between 24 and 48 weeks.^3^ In order to detect clinically appreciable differences, a follow-up of longer than 2 years is suggested.^21^ Another factor to consider is the time to improvement of specific domains of cognitive function. It is well known that HIV primarily impacts functions of subcortical and striatal brain regions, namely executive function, motor and information processing speeds and memory recall.^5^ The IHDS is limited to the evaluation of motor speed, memory recall and psychomotor functions.^22^ In contrast, the BNCE covers a broader range of cognitive functions with a specific focus on executive function.^17^ It is not clear which aspects of cognitive function are first to improve with ART with some short-term studies showing improvement in all domains except motor processing speed.^23^ This is an area requiring further research. Another explanation for the findings could be that high rates of persistent milder forms of neurocognitive impairment are commonly seen despite adequate ART with the BNCE being more appropriate in its recognition.^24^

The BNCE’s advantage over other screening tests is its ability to assess both conventional, frequently used cognitive functioning as well as more advanced levels of executive functioning allowing for detection of milder forms of cognitive impairment.^18^ In assessment of HAND, one can expect Part 1 scores to be relatively preserved and Part 2 scores to show deterioration, with the difference being more pronounced in severe disease.^18^ Results confirmed this in that majority of participants had lower scores in Part 2, confirming the subcortical origin of pathology as well as emphasising the burden of mild neurocognitive impairment based on point discrepancies found between the two parts. In moderate to severe dementia, the score in Part 2 shows earlier and quicker deterioration and with disease progression is expected to remain consistently lower than Part 1 scores.^18^ Interestingly, the results show improvement of BNCE part 2 scores at 3 months and a lower percentage showing the larger discrepancy between the two parts. This trend is in keeping with the subcortical origin of HAND and the quicker deterioration and therefore improvement with treatment in these areas of cognition. Results also show more improvement in Part 2 than Part 1 scores. This confirms that significant improvement in certain areas of cognitive functioning is taking place over the short period examined. However, this does not equate to overall improvement in cognitive functioning.

With the focus of this study on dolutegravir, which when combined in the currently recommended regimen, contributes four points to a total CPE score of seven.^25^ This is considered as having high CNS penetration.^25^ While higher CPE scores are associated with improved control of viral replication in the CNS, its correlation to neurocognitive impairment remains controversial with ongoing concern of neurotoxicity.^25^ Despite this possibility, a recent study concluded that ART with higher CPE is associated with modest improvement in global neurocognitive functioning, with less improvement noted in specific cognitive domains such as attention, working memory and learning.^26^ This could explain why significant improvements were noted in the IHDS and processing speed and not in BNCE with its strong emphasis on higher orders of cognitive function. Overall, it has been found that higher CPE regimens are slightly more likely to preserve or improve cognitive function than regimens with lower CPE scores.^26^ Research comparing dolutegravir-based regimens with other regimens of lower total CPE value would be beneficial.

Cerebrospinal fluid viral escape should also be considered in assessing effects of CPE on neurocognitive function. This occurs when plasma viral suppression is achieved, while CSF viral replication continues.^26^ It occurs in 4% – 20% of patients treated with ART, with some experiencing ongoing neurological symptoms.^26^ This could explain the findings of lack of correlation between neurocognitive improvement and serum VL suppression.

As expected, cognitive test results of BNCE were lower with increasing age. In the ageing HIV population, both accelerated and natural decline can be seen due to multiple factors, with AIDS as the predominant cause and hence expectation is to see a negative effect of age on cognitive function despite adequate treatment.^21^ Evidence also shows that age-related cognitive decline is exacerbated by HIV.^10^ Although the study excluded participants over the age of 60 to account for age-related decline in cognition, some elements of executive function have been found to start declining as early as 45 years of age.^27^ This could also explain why we see a correlation between increasing age and BNCE results and we do not see the same correlation with IHDS results, with its limited assessment of executive function. Another aspect to consider with increasing age is that drug pharmacokinetics may be less effective, negatively impacting the relationship between CPE score and neurocognitive improvement.^26^

The cognitive reserve hypothesis holds that factors such as higher levels of education, socioeconomic status and baseline intelligence can be protective against clinical manifestations of brain pathology.^27^ This study confirmed that for each year increase in years of education, there was an increase in neurocognitive score as well as faster processing speed.

Research regarding gender as a risk factor for neurocognitive impairment is conflicting.^8^ This study did not find any correlation between gender and neurocognitive function; however, this could be affected by over-representation of female participants.

Many large studies have confirmed that a low nadir CD4 is an indicator of neurocognitive impairment as well as a predictor for future diagnosis of HAND.^24,28^ This study confirms that those with lower baseline CD4 counts were more likely to show improved neurocognitive scores and processing speed 3 months after receiving treatment. This could be accounted for by the association of baseline CD4 cell counts of less than 200 cells/mm^3^ with increased proliferation of HIV within the CNS.^29^ Another consideration is the possibility of delirium at baseline prior to the initiation of ART. Risk factors for delirium include the presence of neurocognitive impairment as well as acute and opportunistic infections, which more commonly occur with advanced immunosuppression.^30^ In contrast, multiple studies have found that markers of advanced HIV such as lower CD4 counts were not a significant risk factor for delirium.^30^ The results support early initiation of ART to assist with various degrees of neurocognitive impairment.

This study contributes towards the growing evidence regarding effects of ART on neurocognitive function as well as the need for more evidence around the use of dolutegravir.

### Limitations

Limitations included a small sample size with a significant female predominance, which may have impacted on some results noted. Additionally, the BNCE required adjustments to make the test culturally appropriate. While this is the approach taken in practice and necessary to ensure the suitability of the test to South Africans, construct validity of these changes has not been established. Furthermore, a control group for comparison would have been useful to account for practice effects when interpreting improvements in results. This is an area for further research. It is also acknowledged that complex multifactorial factors contribute towards low cognitive test performance, including socioeconomics, quality of education and comorbidities.^31^ These factors increase the false-positive rate of HAND diagnosis, questioning the validity of the current criteria with a move towards more emphasis on clinical diagnosis.^31^

## Conclusion

Based on the findings, one can conclude that the trend of neurocognitive function is towards improvement in HIV positive, treatment naïve patients who receive 3 months of dolutegravir-based ART. While the dolutegravir cannot be implicated as the main factor contributing towards improvement, the findings support the use of dolutegravir-based regimens in the treatment of patients with HIV-associated neurocognitive impairment. Future research should aim at assessing patients over a longer period to confirm clinically significant improvements. Comparison to other regimens would also be beneficial.

## Data Availability

The data that support the findings of this study are available from the corresponding author, J.R., upon reasonable request.

## References

[1] Saylor D, Dickens AM, Sacktor N, et al. HIV-associated neurocognitive disorder – Pathogenesis and prospects for treatment. Nat Rev Neurol. 2016;12(4):234–248. 10.1038/nrneurol.2016.27 26965674 PMC4937456

[2] Debalkie Animut M, Sorrie MB, Birhanu YW, Teshale MY. High prevalence of neurocognitive disorders observed among adult people living with HIV/AIDS in Southern Ethiopia: A cross-sectional study. PLoS One. 2019;14(3):e0204636. 10.1371/journal.pone.0204636 30883557 PMC6422272

[3] Wang Y, Liu M, Lu Q, et al. Global prevalence and burden of HIV-associated neurocognitive disorder: A meta-analysis. Neurology. 2020;95(19):e2610–e2621. 10.1212/WNL.0000000000010752 32887786

[4] Cross HM, Combrinck MI, Joska JA. HIV-associated neurocognitive disorders: Antiretroviral regimen, central nervous system penetration effectiveness, and cognitive outcomes. S Afr Med J. 2013;103(10):758. 10.7196/SAMJ.6677 24079630

[5] Joska JA, Westgarth-Taylor J, Hoare J, et al. Validity of the International HIV Dementia Scale in South Africa. AIDS Patient Care STDs. 2011;25(2):95–101. 10.1089/apc.2010.0292 21214343

[6] Williams ME, Stein DJ, Joska JA, Naudé PJW. Cerebrospinal fluid immune markers and HIV-associated neurocognitive impairments: A systematic review. J Neuroimmunol. 2021;358:577649. 10.1016/j.jneuroim.2021.577649 34280844

[7] Joska JA, Westgarth-Taylor J, Myer L, et al. Characterization of HIV-associated neurocognitive disorders among individuals starting antiretroviral therapy in South Africa. AIDS Behav. 2011;15(6):1197–1203. 10.1007/s10461-010-9744-6 20614176

[8] Joska JA, Fincham DS, Stein DJ, Paul RH, Seedat S. Clinical correlates of HIV-associated neurocognitive disorders in South Africa. AIDS Behav. 2010;14(2):371–378. 10.1007/s10461-009-9538-x 19326205

[9] Chan LG, Ho MJ, Lin YC, Ong Y, Wong CS. Development of a neurocognitive test battery for HIV-associated neurocognitive disorder (HAND) screening: Suggested solutions for resource-limited clinical settings. AIDS Res Ther. 2019;16(1):9. 10.1186/s12981-019-0224-4 30987670 PMC6463654

[10] Cohen RA, Seider TR, Navia B. HIV effects on age-associated neurocognitive dysfunction: Premature cognitive aging or neurodegenerative disease? Alzheimer’s Res Ther. 2015;7(1):37. 10.1186/s13195-015-0123-4 25848401 PMC4386102

[11] Heaton RK, Clifford DB, Franklin DR, et al. HIV-associated neurocognitive disorders persist in the era of potent antiretroviral therapy: CHARTER Study. Neurology. 2010;75(23):2087–2096. 10.1212/WNL.0b013e318200d727 21135382 PMC2995535

[12] Wei J, Hou J, Su B, et al. The prevalence of frascati-criteria-based HIV-associated neurocognitive disorder (HAND) in HIV-infected adults: A systematic review and meta-analysis. Front Neurol. 2020;11:581346. 10.3389/fneur.2020.581346 33335509 PMC7736554

[13] Ances BM, Letendre SL. CROI 2019: Neurologic complications of HIV disease. Top Antiir Med. 2019;27(1):26–33. PMC6550359 31137000

[14] Republic of South Africa National Department of Health. 2019 ART clinical guidelines [homepage on the Internet]. 2019 [cited 2022 Oct 26]. Available from: https://www.health.gov.za/wp-content/uploads/2020/11/2019-art-guideline.pdf

[15] Chan P, Goh O, Kroon E, et al. Neuropsychiatric outcomes before and after switching to dolutegravir-based therapy in an acute HIV cohort. AIDS Res Ther. 2020;17(1):1. 10.1186/s12981-019-0257-8 31907064 PMC6945418

[16] Meintjes G, Moorhouse MA, Carmona S, et al. Adult antiretroviral therapy guidelines 2017. S Afr J HIV Med. 2017;18(1):776. 10.4102/sajhivmed.v18i1.776 PMC5843236 29568644

[17] Ball TD, Pastore RE, Sollman MJ, Burright RG, Donovick PJ. Evaluation of a neuropsychological screen in an incarcerated population. Clin Neuropsychol. 2009;23(6):1037–1049. 10.1080/13854040902830203 19468963

[18] Tonkonogy J. The brief neuropsychological cognitive examination manual. Los Angeles, CA: Western Psychological Services; 1997.

[19] WMA Declaration of Helsinki – Ethical principles for medical research involving human subjects – WMA – The World Medical Association [homepage on the Internet]. [cited 2023 Feb 25]. Available from: https://www.wma.net/policies-post/wma-declaration-of-helsinki-ethical-principles-for-medical-research-involving-human-subjects/

[20] Adams JL, Choi YC, West M, Pontiggia L, Baxter J, George J. Changes in neurocognitive assessment scores after initiating dolutegravir- versus elvitegravir-based antiretroviral therapy. AIDS Care. 2021;33(11):1507–1513. 10.1080/09540121.2020.1837337 33103919

[21] Damas J, Ledergerber B, Nadin I, et al. Neurocognitive course at 2-year follow-up in a Swiss cohort of people with well-treated HIV. AIDS. 2021;35(15):2469–2480. 10.1097/QAD.0000000000003057 34411034 PMC8631148

[22] Rosca EC, Tadger P, Cornea A, Tudor R, Oancea C, Simu M. International HIV dementia scale for HIV-associated neurocognitive disorders: A systematic review and meta-analysis. Diagnostics. 2021;11(6):1124. 10.3390/diagnostics11061124 34202994 PMC8235728

[23] Zhuang Y, Qiu X, Wang L, Ma Q, et al. Combination antiretroviral therapy improves cognitive performance and functional connectivity in treatment-naïve HIV-infected individuals. J Neurovirol. 2017;23(5):704–712. 10.1007/s13365-017-0553-9 28791662 PMC5655604

[24] Heaton RK, Franklin DR, Ellis RJ, et al. HIV-associated neurocognitive disorders before and during the era of combination antiretroviral therapy: Differences in rates, nature, and predictors. J Neurovirol. 2011;17(1):3–16. 10.1007/s13365-010-0006-1 21174240 PMC3032197

[25] Santos GMA, Locatelli I, Métral M, et al. Cross-sectional and cumulative longitudinal central nervous system penetration effectiveness scores are not associated with neurocognitive impairment in a well treated aging human immunodeficiency virus-positive population in Switzerland. Open Forum Infect Dis. 2019;6(7):ofz277. 31304188 10.1093/ofid/ofz277 PMC6612860

[26] Arentoft A, Troxell K, Alvarez K, et al. HIV antiretroviral medication neuropenetrance and neurocognitive outcomes in HIV+ adults: A review of the literature examining the central nervous system penetration effectiveness score. Viruses. 2022;14(6):1151. 10.3390/v14061151 35746623 PMC9227894

[27] Harada CN, Natelson Love MC, Triebel KL. Normal cognitive aging. Clin Geriatr Med. 2013;29(4):737–752. 10.1016/j.cger.2013.07.002 24094294 PMC4015335

[28] McCombe J, Vivithanaporn P, Gill M, Power C. Predictors of symptomatic HIV-associated neurocognitive disorders in universal health care. HIV Med. 2013;14(2):99–107. 10.1111/j.1468-1293.2012.01043.x 22994556

[29] Azizah N, Machin A, Hamdan M. Low CD4 level increased the risk of cognitive impairment in the HIV patient. Indian J Public Health Res Dev. 2020;11(1):1403–1417. 10.37506/v11/i1/2020/ijphrd/194156

[30] Day C, Manning K, Abdullah F, et al. Delirium in HIV-infected patients admitted to acute medical wards post universal access to antiretrovirals in South Africa. S Afr Med J. 2021;111(10):974–980. 10.7196/SAMJ.2021.v111i10.15628 34949292

[31] Nightingale S, Dreyer AJ, Saylor D, Gisslén M, Winston A, Joska JA. Moving on from HAND: Why we need new criteria for cognitive impairment in persons living with human immunodeficiency virus and a proposed way forward. Clin Infect Dis Off Publ Infect Dis Soc Am. 2021;73(6):1113–1118. 10.1093/cid/ciab366 33904889

